# Discovering heterogeneous treatment effects on slope-based endpoints in chronic kidney disease trials

**DOI:** 10.1186/s12874-025-02646-7

**Published:** 2025-10-14

**Authors:** Tianyu Pan, Lu Tian, Manjula Kurella Tamura, Maria Montez-Rath, Vivek Charu

**Affiliations:** 1 Department of Biomedical Data Science, Stanford University School of Medicine, 1265 Welch Road, Stanford, CA 94305 USA; 2 Department of Pathology, Stanford University School of Medicine, 300 Pasteur Drive, Stanford, CA 94305 USA; 3 Division of Nephrology, Department of Medicine, Stanford University School of Medicine, 3180 Porter Drive, Palo Alto, CA 94304 USA; 4 Veterans Affairs Palo Alto Health Care System, 3801 Miranda Avenue, Palo Alto, CA 94304 USA; 5 Quantitative Sciences Unit, Department of Medicine, Stanford University School of Medicine, 300 Pasteur Drive, Stanford, CA 94305 USA

**Keywords:** Acute and chronic slopes, Bayesian decision tree, Informative censoring, Shared parameter model, Subgroup identification

## Abstract

**Background:**

Chronic kidney disease (CKD) is slowly progressive, with clinically-relevant end-points of interest (e.g. kidney failure, dialysis, transplantation, death due to kidney disease) occurring many years after diagnosis, making the design of trials to evaluate treatments that slow the progression of kidney disease challenging. Recent meta-analyses have shown that the 3-year total slope of estimated glomerular filtration rate (eGFR) may serve as a reliable surrogate for these hard clinical outcomes. Existing research has focused on relaxing the linear trend assumption on the eGFR slope, accounting for informative censoring (via fitting a shared parameter model, for example), and evaluating heterogeneous treatment effects (HTEs) given predetermined subgroups. Yet, none have explored data-driven subgroup identification and HTE estimation.

**Methods:**

We propose a Bayesian method that incorporates a Bayesian decision tree for HTE into a shared-parameter model that combines a survival model for censoring time with a two-slope spline model that characterizes the total eGFR slope. Our proposed approach simultaneously estimates the total eGFR slope in the presence of informative censoring and identifies interpretable subgroups of patients who experience differential treatment effects on the total eGFR slope outcome.

**Results:**

Simulation studies demonstrate that our method accurately recovers treatment-effect heterogeneity with low estimation error, yielding better subgroup-specific treatment recommendations in moderate-to-large samples. Our method also controls false positives when no true heterogeneity presents. We apply our approach to the Modification of Diet in Renal Disease (MDRD) Trial, observing strong Bayesian evidence that patients with a baseline eGFR above 34.32 benefit more from the intensive systolic blood pressure control compared to patients with a baseline eGFR below 34.32. Specifically, the posterior probability that the treatment effect is larger in the higher-eGFR subgroup is 81 %.

**Conclusion:**

Our proposed model can effectively capture even subtle HTEs while avoiding over-fitting when no heterogeneity exists, making it valuable for identifying HTE to inform downstream analyses such as treatment recommendations.

**Supplementary Information:**

The online version contains supplementary material available at 10.1186/s12874-025-02646-7.

## Introduction

Chronic kidney disease (CKD) typically progresses slowly over extended periods of time. In patients with early-stage CKD, hard clinical endpoints, such as end-stage kidney disease (ESKD), accrue infrequently, and as such, clinical trials to evaluate the effect of treatments to slow the progression of CKD in these populations require extended follow-up and large sample sizes. For example, under a 1:1 randomization design, detecting a 20% reduction in in hazard (hazard ratio (HR) = 0.8) with 80% power requires observing approximately 631 events [1, 2], which, in a population of patients with early-stage CKD, can translate into a trial requiring decades of follow-up.

To address these constraints, the 3-year total slope in estimated glomerular filtration rate (eGFR), has emerged as reliable surrogate of treatment effects on clinical endpoints: meta-analyses report median $R^2$ of 0.97 for the association between treatment effects on the 3-year eGFR slope, estimated via shared parameter models, and those on the time to a clinical kidney endpoint (doubling of serum creatinine, GFR<15 ml/min per 1.73 $m^2$ or kidney failure with replacement therapy), measured by HR from Cox models [3, 4]. Employing the 3-year eGFR slope permits shorter assessment windows and reduces reliance on rare kidney events, hence enhancing statistical power and trial efficiency, especially in early-stage CKD populations.

Estimation of the total eGFR slope requires consideration of informative censoring/non-ignorable dropout. For patients who transition to dialysis, the measured eGFR no longer accurately reflects their true GFR; for patients who die (even from non-renal causes), eGFR measurements are not possible [5–7]. Consequently, if the time-to-dropout is associated with the patients’ eGFR trajectories, e.g., patients with rapidly declining eGFR are more likely to drop out, traditional methods that fail to account for this mechanism can lead to invalid inferences [7, 8]. To tackle this issue, the shared parameter (SP) model is a well-known strategy that helps mitigate bias derived from such dropout mechanisms [9].

Despite use of shared-parameter (SP) models to estimate average treatment effects on the total eGFR slope, existing approaches have not fully explored how to best identify its heterogeneous treatment effects. Importantly, prior work has found no significant variation in the performance of the total eGFR slope surrogate outcome across subgroups defined by baseline GFR, UACR, cause of disease, and control-arm decline [10, 11]. This absence of surrogacy heterogeneity justifies the development of methods to identify heterogeneous treatment effects on the total eGFR slope outcome.

A common approach to characterize HTE is to evaluate treatment effects within pre-specified subgroups based on clinical experience. This approach can miss meaningful treatment effect heterogeneity in clinically unrecognized subgroups, and can suffer from false positive results due to multiple testing [12]. In longitudinal studies, a promising solution is to develop methods that generate data-driven sub-grouping criteria, often through constructing decision trees. This approach has gained increasing attention in recent years, especially for discovering HTE in longitudinal data using tree-based methods [13–15]. However, these methods deal with a simpler setting without informative censoring. We develop a decision tree-based method [16, 17] built on the two-slope Shared Parameter (SP) model structure [1], which addresses possible informative censoring. Specifically, in the SP model specification, we allow the baseline eGFR, the acute and chronic eGFR slopes in the control arm, and the chronic and conditional average treatment effects on the eGFR slope to depend on baseline covariates. These relationships are modeled by Bayesian decision trees, which approximate the underlying unknown functions and readily provide subgroup structure due to their discrete nature. Our method effectively adjusts the potential informative censoring, captures possible HTEs, and produces meaningful clinical subgroups.

This paper is organized as follows. In “Method” section, we introduce the specifications of the two-slope SP model and Bayesian decision trees. In “Model” section, we present our model as an integration of Bayesian decision trees with the two-slope SP model, along with model fitting details. In “Simulation” section we present simulation studies investigating the validity and sensitivity of our model. Finally, in “MDRD data analysis” section, we apply our model to reanalyze the Modification of Diet in Renal Disease dataset (MDRD; [18, 19]).

## Method

In this section, we introduce our proposed model, which consists of three key components: a two-slope linear spline model to capture the treatment effect on the total eGFR slope, a SP model to account for possible informative censoring, and a Bayesian decision tree (BDT) to identify the HTEs.

### A two-slope concatenated spline model for eGFR

In CKD clinical trials, the change of eGFR is often non-linear after the initiation of an intervention [1, 3, 4]. Specifically, patients can experience an acute decline in eGFR during the initial period after treatment [3], followed by a slower decline over a longer period of time. Throughout the rest of this paper, we will refer to these two rates as the acute slope and the chronic slope, respectively.

To capture this non-linearity, existing methods suggest modeling the acute and chronic slopes separately using two concatenated linear regression models, with a “change-point” at a predefined time knot [1, 3]. In this paper, we propose using a two-slope linear spline model to capture the mean trend of eGFR trajectories by treatment arm. Additionally, we incorporate a mixed-effect structure to account for within-subject correlations. Suppose $Y_{ij}$ is the observed *j*th eGFR level recorded at year $\text {yrs}_{ij}$ since baseline for subject *i*, $\text {trt}_i$ is the binary treatment indicator, $t^*$ is a pre-specified time knot, and $d_{ij}\equiv \mathbbm{1}(\text {yrs}_{ij}\ge t^*)$ is an indicator for modelling the two-slope nature of eGFR decline, where $d_{ij}=1$ refers to the chronic period. The model is encapsulated as follows,

1 $$\begin{aligned} & Y_{ij} = \mu _{ij} + \epsilon _{ij},~\text {for}~j=1,\ldots ,n_i,~i=1,\ldots ,n,\nonumber \\ & \mu _{ij} = \underbrace{(\alpha _{0} + \alpha _i)}_{\text {intercept}} + \underbrace{(\beta _0 + \beta _i)}_{\text {acute slope}} \times (\text {yrs}_{ij} - \text {d}_{ij}\times (\text {yrs}_{ij} - t^*)) +\nonumber \\ & \underbrace{(\gamma _0 + \gamma _i)}_{\text {chronic slope}} \times d_{ij} \times (\text {yrs}_{ij} - t^*) +\nonumber \\ & \underbrace{\zeta _0}_{\text {acute treatment effect}} \times \text {trt}_i \times (\text {yrs}_{ij} - \text {d}_{ij}\times (\text {yrs}_{ij} - t^*)) +\nonumber \\ & \underbrace{\nu _0}_{\text {chronic treatment effect}} \times \text {trt}_i \times d_{ij} \times (\text {yrs}_{ij} - t^*). \end{aligned}$$

In this model, $\alpha _0$ denotes the expected baseline eGFR, $\beta _0$ denotes the expected acute slope in the control group, and $\zeta _0$ denotes the acute treatment effect. Similarly, $\gamma _0$ denotes the expected chronic slope in the control group, and $\nu _0$ denotes the chronic treatment effect. While $\epsilon _{ij}$ is an independent random error, $\alpha _i$, $\beta _i$, and $\gamma _i$ are subject-specific random effects. Here, we assume that $\epsilon _{ij}\sim N(0, \sigma ^2),$ and the subject-specific random effect $(\alpha _i,\beta _i,\gamma _i)^{T} \sim N(0,\sigma ^2\times \Sigma ).$

The current model specification ensures that the two linear regression models are concatenated at $t^*$. In clinical studies, $t^*$ is often set to 1/3, i.e., the fourth month after the intervention [3, 4], or to coincide with the first study visit after randomization [1]. In our analysis of MDRD, we fix $t^* = 1/3$ to ensure that at least two records per subject (one at baseline and one at first follow up) contribute to the estimation of the acute slope.

In addition to the acute and chronic slopes, clinicians are interested in evaluating the overall efficacy of a treatment in reducing the decline of eGFR slope over a given time range. The integrative treatment effect on the acute and chronic slopes, named the total treatment effect [1], is defined as a weighted average,

2 $$\begin{aligned} \tau _0= & w\times \zeta _0 + (1 - w) \times \nu _0,\nonumber \\ w= & \frac{t^*}{T^*}, \end{aligned}$$

where $T^*$ represents a pre-specified time frame, which can be either a pre-determined value when a trial is proposed, or an administrative censoring time after a trial is completed. In our analysis of MDRD, we choose $T^*=3$ years, inspired by the conclusions of a recent meta-analysis [3, 4], indicating that the treatment effect on the 3-year total eGFR slope is an effective surrogate for clinical endpoints in patients with CKD.

By substituting $\nu _0$ in (1) with $\tau _0$, the equation can be reparametrized in terms of the total treatment effect $\tau _0$ defined in (2). The reparametrized format will be adopted for the following simulation tasks and real data analyses.

### A shared parameter model structure for informative censoring

In CKD trials, the eGFR slope is associated with progression to terminal CKD events that preclude further observation of eGFR. This informative censoring mechanism poses challenges in estimating the eGFR slope. An accepted remedy is to use a SP model [9, 20]. Briefly, a SP model leverages the information from a time to event outcomes, such as the time to death or the onset of CKD-specific events such as (e.g. kidney failure requiring transplantation or dialysis), to help infer the subject-level random effects quantifying the eGFR trajectory and estimate fixed effects of interest.

To model the time to event outcomes in our study, we employ a piecewise exponential model for its flexibility [21]. The model is detailed as follows,

3 $$\begin{aligned} S_i(t)= & \exp \left\{ -\int _0^t z_i\times h(s) ds\right\} ,\nonumber \\ z_i= & \exp \left\{ \lambda _0\times \text {trt}_i + \eta _{1}\times \alpha _i + \eta _{2}\times \gamma _i\right\} ,\nonumber \\ h(s)= & \exp \left\{ \sum \limits _{k=1}^K\lambda _k \times \mathbbm{1}(s\in [t_k,t_{k+1}))\right\} , \end{aligned}$$

where $S_i(t)$ denotes the survival function of the *i*-th subject, *h*(*t*) is the baseline hazard function, $\lambda _0$ represents the treatment effect on the time to event outcome, and $\eta _1$ and $\eta _2$ are the connecting parameters that join the longitudinal and survival models. The random effects $(\alpha _i,\gamma _i)$ are the same as those in (1). The number of steps *K* is chosen to be 5, as it exhibits sufficient flexibility, with pre-fixed time knots $\{t_k\}_{k=1}^{K+1}$ placed at the quantiles of the observed event times. The event time $T_i$ is defined as the time to a composite endpoint, consisting of (1) all-cause mortality, and (2) end-stage kidney disease (ESKD; inclusive of initiation of dialysis and transplantation) for the MDRD data analysis.

#### Remark 1

There are some possible alternative formulations of (3) that are not adopted here. First, the acute random effect $\beta _i$ is intentionally excluded from $z_i$. During the acute stage, very few eGFR values are recorded for each subject and there is little information about $\beta _i$, much less its association with the event time. Second, one may generalize the formulation of $z_i$ in (3) to include baseline or even time-varying covariates, but this is not recommended due to the concern on identifiability and slow mixing in the corresponding Bayesian computation.

### Heterogeneous treatment effect detection with Bayesian decision trees

Understanding HTE on slope-based endpoints has received increasing attention [3, 4]. Traditionally, the identification of subgroups for downstream analyses is based on clinical insight and is restricted to pre-defined subgroups. In this section, we present a data-driven approach using BDT [16, 17], which infer decision rules for generating subgroups with HTE.

For notation clarity, we revise the intercept $\alpha _0$, the acute slope $\beta _0$, the chronic slope $\gamma _0$, the total treatment effect $\tau _0$ and the acute treatment effect $\zeta _0$ defined in (1) and (2) into functions of covariates *x* : $\alpha _{0}(x)$, $\beta _{0}(x)$, $\gamma _{0}(x)$, $\tau _{0}(x)$ and $\zeta _0(x)$ using BDTs,

4 $$\begin{aligned} & \alpha _{0}(x_i) = x_i^T\mu _{\mathcal{B}\mathcal{T}_0(x_i)}^{(0)},~~\gamma _{0}(x_i) = x_i^T\mu _{\mathcal{B}\mathcal{T}_1(x_i)}^{(1)},~~\tau _{0}(x_i) = \theta _{\mathcal{B}\mathcal{T}_2(x_i)}^{(2)}, \nonumber \\ & \beta _0(x_i) = x_i^T\mu ^{(3)},~~\zeta _0(x_i) = x_i^T\mu ^{(4)}, \nonumber \\ & \mathcal{B}\mathcal{T}_k(x): \mathbbm{R}^{p} \rightarrow \{1, \cdots , J_k\}\sim P(c_0,d_0),~\text {for}~k~\in \{0, 1, 2\}, \nonumber \\ & \mu _{j}^{(k)} \sim N(0,\tau _0^2I_{p\times p})~\text {for}~j\in \{1, \cdots , J_k\}~\text {and}~k \in \{0,1\}, \nonumber \\ & \theta _{j}^{(2)} \sim N(0,\tau _0^2),~\text {for}~ j\in \{1, \cdots , J_2\}, \nonumber \\ & \mu ^{(3)}, \mu ^{(4)}\sim N(0,\tau _0^2I_{p\times p}), \end{aligned}$$

where $$x_i$$ is a vector of length *p* including the intercept, which denotes the baseline covariate of the *i*-th subject, $$\mathcal{B}\mathcal{T}_k(\cdot )$$ denotes a binary decision tree generated from a prior $$P(c_0,d_0)$$ with “birth” and “death” proposals as introduced in [16], $$\mu _1^{(k)}, \cdots , \mu _{J_k}^{(k)}$$ are $$J_k$$ column vectors of length *p*, representing the within-leaf linear regression coefficients for $$k=0, 1,$$$$\theta _1^{(2)}, \cdots \theta _{J_2}^{(2)}$$ are $$J_2$$ scalars, representing the within-leaf average treatment effect on total slope, and $$\mu ^{(3)}$$ and $$\mu ^{(4)}$$ are column vectors of length *p*, representing the linear regression coefficients for the acute slope and acute treatment effect in the entire population, respectively. For convenience, we denote $$\left\{\mu _1^{(k)}, \cdots , \mu _{J_k}^{(k)}\right\}$$ and $$\left\{\theta _1^{(2)}, \cdots , \theta _{J_2}^{(2)}\right\}$$ by $$\mu ^{(k)}_{\mathcal{B}\mathcal{T}_k(\cdot )}$$ and $$\theta ^{(2)}_{\mathcal{B}\mathcal{T}_2(\cdot )},$$ respectively. The parameters $$c_0$$ and $$d_0$$, named the base and power parameters, control the depth of the generated trees. A decision tree can be viewed as a piece wise constant function dividing covariate spaces into different subregions defined by a sequence of binary rules. For our purpose, patients from a “leaf node” in a decision tree consist an identified sub-population. In theory, a BDT can be of any depth, allowing it to capture all possible sub-populations without any prior knowledge of the number of sub-populations. However, the third line of (4) features a Bayesian tree generating mechanism that naturally controls the number of identified sub-populations through Bayesian penalties on the depth of the generated tree. This helps avoid the discovery of spurious small sub-populations and mitigate the inflation of Type I errors that underlie the multiple-testing problem [22, 23].

**Fig. 1:**
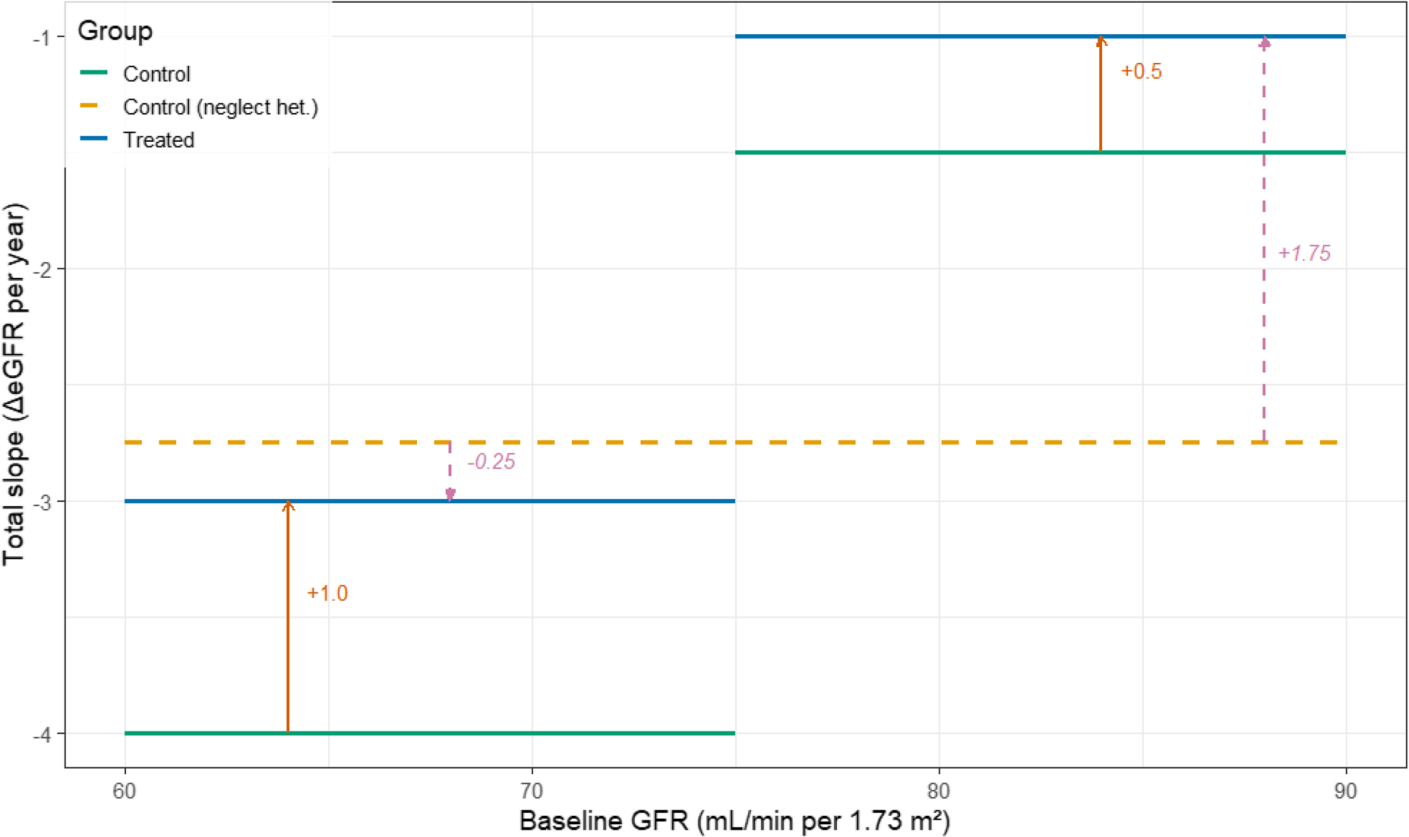
Treatment effect estimations can be misleading if the heterogeneity in the control group slope is overlooked

Next, we provide interpretations and rationales for the formulation (4). First, in addition to the tree structure imposed on the total treatment effect $\tau _0(\cdot )$, we also assume that the intercept and the chronic slope in the control arm are modeled by linear regression models within the leaf nodes induced by the respective decision trees: $\alpha _0(x)=x^T \mu ^{(0)}_{\mathcal{B}\mathcal{T}_0(x)}$ and $\gamma _0(x)=x^T \mu ^{(1)}_{\mathcal{B}\mathcal{T}_1(x)}$. Compared to assuming a constant intercept and slope, this modeling strategy allows flexible covariate effect on the intercept and the chronic slope in the control arm. Underfitting the chronic slope, which constitutes the majority of the total slope, might lead to misleading conclusions about treatment efficacy. In Fig. 1, a toy example illustrates that ignoring heterogeneity in the control-group slope can spuriously suggest a negative HTE among patients with baseline GFR $<75$ (pink arrow), even though the true treatment effect is positive for all patients.

Second, the tree-based partitioning of the covariate space induces a piecewise-constant treatment effect:

5 $$\begin{aligned} \tau _0(x) =\sum _{j=1}^{J_2}\theta ^{(2)}_j\,\mathbbm{1}\{\mathcal{B}\mathcal{T}_2(x)=j\}. \end{aligned}$$

which can be characterized via a set of simple binary decision rules. For example, in Fig. 1, properly accounting for heterogeneity in the control and treatment slopes yields

$$\begin{aligned} \text{ treatment } \text{ slope }= & -3\times \mathbbm{1}\{\text {Baseline GFR}<75\}-1\times \mathbbm{1}\{\text {Baseline GFR}\ge 75\},\\ \text{ control } \text{ slope }= & -4\times \mathbbm{1}\{\text {Baseline GFR}<75\}-1.5\times \mathbbm{1}\{\text {Baseline GFR}\ge 75\}, \end{aligned}$$

and

6 $$\begin{aligned} \tau _0(\text {Baseline GFR})= & 1\times \mathbbm{1}\{\text {Baseline GFR}<75\}+\nonumber \\ & 0.5\times \mathbbm{1}\{\text {Baseline GFR}\ge 75\}, \end{aligned}$$

indicating that patients with lower baseline GFR receive greater benefit. In real applications, the control and treatment total slopes as functions of baseline covariates such as urine albumin-tocreatinine ratio (UACR) and age may be very different and induce a complicated functional form for $\tau _0(\cdot ).$ Nonetheless, modeling $\tau _0(x)$ as a piece-wise constant function in (5) ensures the identification of simple subgroups of patients and the corresponding subgroup-specific treatment effects.

Third, tree structures are not considered for the acute slope and acute treatment effect, due to a paucity of information about their individual-level random effects, as explained in the last paragraph of “A shared parameter model structure for informative censoring” section. Empirically, we notice that flexible tree structure, if also imposed on $\beta _0(x)$ and $\zeta _0(x)$, often results in a single root node tree likely due to a low signal-to-noise ratio. Therefore, we select simple linear functions for modelling $\beta _0(x)$ and $\zeta _0(x)$ in our study for modelling and computational convenience.

## Model

### Bayesian hierarchical structure

In this section, we formally present the Bayesian hierarchical structure of our proposed model. The notations used are consistent with those introduced in “Method” section. For clarity, we present the definition and the modeling strategy of the used parameters in Table 1 and 2. Our model is detailed as follows in (7),

7 $$\begin{aligned} {\textbf {(1) }} & {\textbf {Two-slope concatenated spline:}}\nonumber \\ & Y_{ij}\mid \mu _{ij},\{\mathcal{B}\mathcal{T}_j(\cdot )\}_{j=0}^2{\mathop {\sim }\limits ^{ind}} N(\mu _{ij},\sigma ^2),\nonumber \\ & \mu _{ij} = (\alpha _{0}(x_i) + \alpha _{i}) + (\beta _0(x_i) + \beta _{i})\times (\text {yrs}_{ij} - \text {d}_{ij} \times (\text {yrs}_{ij} - t^*)) +\nonumber \\ & (\gamma _{0}(x_i) + \gamma _i)\times \text {d}_{ij}\times (\text {yrs}_{ij} - t^*)+\nonumber \\ & \tau _{0}(x_i) \times \text {trt}_i \times \text {d}_{ij} \times (\text {yrs}_{ij} - t^*)\times \frac{1}{1 - w}+\nonumber \\ & \zeta _0(x_i) \times \text {trt}_i\times \left( \text {yrs}_{ij} - \frac{1}{1 - w}\times d_{ij}\times (\text {yrs}_{ij} - t^*)\right) ,\nonumber \\ & \text {d}_{ij}\equiv \mathbbm{1}(\text {yrs}_{ij}> t^*),\nonumber \\ & (\alpha _i,\beta _i,\gamma _i)^{T}\mid \sigma ^2, \Sigma {\mathop {\sim }\limits ^{i.i.d.}} N(0,\sigma ^2\times \Sigma ), \end{aligned}$$

$$\begin{aligned} {\textbf {(2) }} & {\textbf {Survival function of the i-th subject:}}\\ & S_i(t) = \exp \left\{ -\int _0^t z_i\times h(s) ds\right\} ,\\ & z_i = \exp \left\{ \lambda _0\times \text {trt}_i + \eta _{1}\times \alpha _i + \eta _{2}\times \gamma _i\right\} ,\\ & h(s) = \exp \left\{ \sum _{k=1}^K\lambda _k \times \mathbbm{1}(s\in [t_k,t_{k+1}))\right\} , \end{aligned}$$

$$\begin{aligned} {\textbf {(3) }} & {\textbf {Tree structure:}}\\ & \alpha _{0}(x_i) = x_i^T\mu _{\mathcal{B}\mathcal{T}_0(x_i)}^{(0)},~~\gamma _{0}(x_i) = x_i^T\mu _{\mathcal{B}\mathcal{T}_1(x_i)}^{(1)},~~\tau _{0}(x_i) = \theta _{\mathcal{B}\mathcal{T}_2(x_i)}^{(2)},\\ & \beta _0(x_i) = x_i^T\mu ^{(3)},~~\zeta _0(x_i) = x_i^T\mu ^{(4)}, \end{aligned}$$

with the prior specified below,

8 $$\begin{aligned} {\textbf {(4) }} & {\textbf {Priors:}} \nonumber \\ & \{\lambda _i\}_{i=1}^K,\eta _{1},\eta _{2} \sim \mathcal {N}(0,\tau _0^2), \nonumber \\ & \mathcal{B}\mathcal{T}_k(x): \mathbbm{R}^{p} \rightarrow \{1, \cdots , J_k\}\sim P(c_0,d_0),~\text {for}~k~\in \{0, 1, 2\}, \nonumber \\ & \mu _{j}^{(k)} \sim N(0,\tau _0^2I_{p\times p})~\text {for}~j\in \{1, \cdots , J_k\}~\text {and}~k \in \{0,1\}, \nonumber \\ & \theta _{j}^{(2)} \sim N(0,\tau _0^2),~\text {for}~ j\in \{1, \cdots , J_2\},\nonumber \\ & \sigma ^{-2}\sim \text {Gamma}(a_0,b_0), ~\Sigma ^{-1}\sim \mathcal {W}(2 + a_0,b_0^{-1}\times I_{2\times 2}), \end{aligned}$$

**Table 1:** Variables used in the Bayesian hierachical model

Variable	Definition	Modeling strategy
$\alpha _{0}(x)$	The expected baseline eGFR.	Within-leaf linear
		regression
$\beta _0(x)$	The acute slope in the control arm.	Linear regression
$\gamma _{0}(x)$	The chronic slope in the control arm.	Within-leaf linear
		regression
$\tau _{0}(x)$	The treatment effect on the total slope.	Within-leaf linear
		regression
$\zeta _0(x)$	The treatment effect on the acute slope.	Linear regression
$(\alpha _i,\beta _i,\gamma _i)$	The subject specific random effects of	Multivariate normal
	$(\alpha _{0}(x_i), \beta _{0}(x_i), \gamma _{0}(x_i))$.	distribution,
		$$N(0,\sigma ^2\times \Sigma )$$.
$\sigma ^2$	The within-subject variance.	Inverse-Gamma
		distribution
$\Sigma$	The standardized random effect	Inverse-Wishart
		distribution
	covariance using the	
	within-subject variance.	
$\lambda _0$	The treatment effect	Normal distribution
	on the time to event outcome.	
$\{\lambda _i\}_{i=1}^K$	The step values defined in the	
	stepwise baseline hazard function.	
$(\eta _1,\eta _2)$	The connecting parameters	
	in the SP model.	

**Table 2:** Variables used in the Bayesian hierachical model (cont’d)

Variable	Definition	Modeling strategy
$\mathcal{B}\mathcal{T}_0(\cdot ),\mathcal{B}\mathcal{T}_1(\cdot ),\mathcal{B}\mathcal{T}_2(\cdot )$	The decision trees imposed on	Prior introduced in
		[16].
	the expected baseline eGFR,	
	the chronic slope among controls,	
	and the total treatment effect.	
$\mu _{\mathcal{B}\mathcal{T}_0(\cdot )}^{(0)}, \mu _{\mathcal{B}\mathcal{T}_1(\cdot )}^{(1)}$	Within-leaf linear regression	Each within-leaf
	coefficients of $\alpha _0(x)$ and $\gamma _0(x)$.	coefficient follows a
		multivariate normal
		distribution
$\mu ^{(3)}$, $\mu ^{(4)}$	Regression coefficients of the acute	Multivariate normal
	slope among controls, i.e., $\beta _0(x),$	distribution
	and acute treatment effect, i.e., $\zeta _0(x)$.	
$\theta _{\mathcal{B}\mathcal{T}_2(\cdot )}^{(2)}$	Within-leaf mean parameter of	Each follows a
	the total treatment effect tree $\mathcal{B}\mathcal{T}_2(\cdot )$	normal distribution

For the hyper-parameter setting, our principle is to use vague priors due to the lack of prior knowledge. Specifically, we choose $\tau _0 = 1,000$, $a_0 = 0.01$ and $b_0 = 0.01$ to maintain sufficient non-informativeness, allowing data-driven results. The base and power parameters of the tree-generating process are set to $c_0 = 0.95$ and $d_0 = 2$ following the suggestions by [16]. Our empirical results indicate that these hyper-parameter settings effectively generate trees with a moderate number of leaf nodes (sub-populations) and capture subtle HTE, as further supported by simulation results in “Simulation” section.

### Implementation

The proposed model operates as a “two-stage procedure”. In the first stage, a tree-generating process is applied to identify possible subgroups, each representing a sub-population with a distinct treatment effect on the eGFR total slope. In the second stage, a conventional SP model with associated subject specific random effects is fitted for each subgroup identified in the first stage. Through Gibbs sampling, our model integrates these two stages, ensuring valid posterior inferences and addressing potential overfitting of the decision tree using Bayesian prior penalties, as detailed in “Heterogeneous treatment effect detection with Bayesian decision trees” section.

Specifically, we use a Gibbs sampler that iterates between aforementioned two main steps to generate the BDT and the SP samples using their full conditional distributions. In the first step, conditional on the current BDTs, denoted as $\mathcal{B}\mathcal{T}_0(\cdot )$, $\mathcal{B}\mathcal{T}_1(\cdot )$, $\mathcal{B}\mathcal{T}_2(\cdot )$, and the associated regression coefficients $\mu ^{(0)}_{\mathcal{B}\mathcal{T}_0(\cdot )},\mu ^{(1)}_{\mathcal{B}\mathcal{T}_1(\cdot )},\theta ^{(2)}_{\mathcal{B}\mathcal{T}_2(\cdot )}$, we draw posterior samples for SP model parameters, i.e., $\eta _1,\eta _2$, and $\{\lambda _i\}_{i=0}^K$. In the second step, we reverse the procedure, conditional on current SP model parameters to draw posteriors samples for BDTs and associated regression coefficients in the two-slope model. A posterior sample from model (7) is thus obtained after one “round-trip” of these two steps.

Within each “round-trip” iteration, we used the Metropolis–Hastings algorithm to sample from the conditional distribution. To ensure the quality of the posterior samples, we generated a MCMC of length $B_1=100,$ keeping only the last sample, which is expected to approximate the corresponding full conditional distribution better than the initial draw. Sampling BDTs conditional on SP model parameters is realized using the algorithm by [16], which employs “birth” and “death” proposals for the Metropolis–Hastings algorithm. Sampling SP model parameters conditional on BDTs is realized using Rstan with the default “No-U-Turn” sampler [24, 25], which leverages the gradient information of the posterior density surface with adaptive path lengths. The $B_1$ MCMC iterations are halved into “Warmup” and “Sampling” stages in Rstan for model fitting.

After multiple “round-trips”, $B_2=100$ different decision trees are generated, which poses difficulty for summarizing the results since typical metrics, such as mean and median, are inapplicable for trees. One possible approach is to use the “minimum distance tree” as the “most representative tree” [26], i.e., selecting the tree whose posterior conditional average total treatment effect estimation is closest to the posterior mean of $\tau _0(x)$. Although this method is criticized for producing non-unique results, the resulting tree still reliably estimates the total treatment effect. In our study, it is not crucial for the summarized decision tree (clinical decision rules) to be unique. The primary requirement is that it effectively facilitates the subgroup identification based on HTE.

A potential concern arises when studying the interval estimates of the treatment effects given the detected tree structures. BDT is likely to propose a split increasing the difference in estimated treatment effects between two child nodes. Such a procedure suffers from the overfitting bias, inducing the under-coverage of generated credible intervals for the true average treatment effect in each node. To handle this concern, we adopt “honest” trees construction [27, 28], where the dataset is divided into two parts: the first part is used for discovering tree structures (discovery set), and the second part is kept for the honest treatment effect estimation given the tree structure (validation set). For both simulation and real data analyses, we split the data 60/40, using the first $60\%$ data for tree discovery and the remainder for the treatment effect estimation. Operationally, after obtaining the “most representative trees” for the intercept and chronic slope in the control group and the total treatment effect based on the discovery set, a SP model including the two-slope spline regression model for eGFR trajectories is refitted based on the remaining validation set given the tree structures to make inference on the average treatment effects within each leaf node.

To account for the randomness in data splitting, we may repeat the aforementioned analyses a large number of times, e.g., $B_3=100$ times, and yield $B_3$ “most representative trees” for HTE. We then select a super-representative tree from these $B_3$ “most representative trees” to guide the individualized treatment recommendation. There is no unique way to select this super-representative tree. One simple approach is to select the tree that appears the most frequently among $B_3$ generated “most representative trees”. This is possible when we restrict the number of candidate BDT configurations by fixing the potential cut-offs a priori. In other words, the cardinality of the tree space becomes finite. In the example section, we will provide more details for this approach. The entire implementation pipeline of our approach is summarized in Figs. 4 and 5 in the Supplementary File.

#### Remark 2

Sampling the tree structures is challenging due to the absence of a closed-form sampler and hence requires further clarification. One possible solution is to augment the parameter space by additionally sampling the individual-wise random effects $\{\alpha _i,\beta _i,\gamma _i\}_{i=1}^n$ in the second step. The merit is to allow a closed-form evaluation of the marginal likelihood, which facilitates the tree sampling. However, this approach may fail to capture subtle HTE with a reasonable number of Gibbs iterations, as random effects $\{\alpha _i,\beta _i,\gamma _i\}_{i=1}^n$ are “coarse” due to their stochastic nature. This drawback is significant, especially when the average treatment effect on the 3-year total slope is small relative to noise level, as suggested by Table 6 of [1]. Empirical results from our simulations suggest when the difference in the 3-year treatment effect across sub-populations is subtle and small, the parameter augmentation strategy fails to detect the underlying HTE. To tackle this problem, we bypass the individual-wise parameter sampling through marginalization. This approach, however, poses the challenge of losing the closed-form likelihood evaluation used in the sampling scheme. To mitigate this, we employ Laplace’s approximation [29], which approximates the product likelihood of the survival outcome likelihood and the prior of the individual-wise parameters using a multivariate normal distribution. Although this provides a rough approximation of the individual-wise parameter distribution, Laplace’s approximation facilitates the computation of the marginal likelihood for tree sampling by maintaining the likelihood-prior conjugacy. Our simulation results indicate that Laplace’s approximation does not significantly distort the posterior surface, allowing the model to correctly capture HTE, even when the heterogeneity is small.

#### Remark 3

Once subgroups are identified via our approach, re-fitting conventional SP models within these subgroups for inference on the conditional average treatment effects requires caution. This is because the estimated BDT $\mathcal{B}\mathcal{T}_2(\cdot )$ does not characterize the possible nonlinear covariate effect on the intercept and chronic slope in the control arm, and this potential dependence would not be captured in a conventional SP model. As demonstrated by the toy example in Fig. 1, mis-specifying them may lead to biased inference on the total treatment effect. To estimate the conditional average treatment effect, one may fit the conventional SP model in subgroup defined via the intersection of trees constructed for the intercept $\alpha _0(\cdot )$, the chronic slope in the control arm $\gamma _0(\cdot )$ and the total treatment effect $\tau _0(\cdot )$. The detailed procedure for generating an intersection tree is provided in the Supplementary File. The intersection tree tends to have smaller nodes than the tree approximating $\tau _0(\cdot )$ only, rendering possible difficulties in fitting SP models within each node. Despite this, the tree $\mathcal{B}\mathcal{T}_2(\cdot )$ can still be used for treatment recommendations, and the treatment effects derived from the model (which includes potential dependencies between baseline covariates and the control chronic slope and the intercept) are valid estimates of the conditional average treatment effects.

## Simulation

In this section, we evaluate our proposed approach under scenarios with heterogeneous treatment effects depending on both continuous and binary covariates. Our data-generating processes are designed through the following steps based on the model introduced in (7) and the definitions given in (1) and (2). First, we consider six covariates and let the first five be generated from $\text {Unif}(-2,2)$ and the last one from $\text {Bernoulli}(0.5)$. Second, the treatment effects are designed under both true and wrong models. Specifically, the conditional average total treatment effects are modeled as either step (ST) or continuous (CON) functions over the covariates space. These functions depend only on the first (continuous) and sixth (binary) covariates, while the other covariates are included to test the ability of our model to handle nuisance predictors. Third, the intercept and chronic slope terms are assumed to follow similar functional forms as the total treatment effects throughout the simulations. Fourth, the acute slope and its treatment effect are assumed to be peice-wise constant (CST) the covariate space Fifth, different informative censoring (**IC**) levels are explored by choosing $\eta _1,\eta _2$ to be 0 (NO) or −0.5 (YES). The latter scenario represents the situation where patients with lower initial eGFR and faster declination in eGFR are more likely to drop out from the study.

The performance of our model is evaluated from two perspectives using 16 different settings for data generation. In settings 1-8, we assess the model’s ability to identify the total treatment effect heterogeneity and to provide precise treatment recommendations, compared to a baseline approach that treats all patients the same (given a positive average treatment effect, for example). These abilities are measured using three metrics,

9 $$\begin{aligned} \ell _1= & \frac{1}{n_2}\sum _{i=1}^{n_2}\left| \hat{\tau }_{0}(x_i) - \tau _0(x_i)\right| ,\nonumber \\ \text {R}_1= & \frac{1}{n_2}\sum _{i=1}^{n_2}\left| \tau _0(x_i)\right| \times \left| \mathbbm{1}(\hat{\tau }_{0}(x_i)> 0) - \mathbbm{1}(\tau _0(x_i)> 0)\right| ,\nonumber \\ \text {Htr}= & \left| \mathbbm{1}(\hat{\tau }_{0}(x_1) = \ldots = \hat{\tau }_{0}(x_{n_2})) - \mathbbm{1}(\tau _0(x_1) = \ldots = \tau _0(x_{n_2}))\right| , \end{aligned}$$

where $\{x_1, \cdots , x_{n_2}\}$ represents the collection of covariate vectors in the validation set of size $n_2=0.4n$, $\tau _0(\cdot )$ is the true conditional average treatment effect, $\hat{\tau }_0(\cdot )$ is the estimator of $\tau _0(\cdot )$ using our method, $\ell _1$ measures the accuracy of estimating the total treatment effect on the total eGFR slope, $\text {R}_1$ evaluates the loss of adopting the treatment recommendation based on the posterior estimation, compared to the oracle best, $\text {Htr}$ indicates whether our model correctly identifies the presence of heterogeneity, with $\text {Htr} = 0$ representing correct identification and 1 otherwise. To further interpret these metrics in the context of estimating the total slope, we can interpret the estimates $\ell _1$, $\text {R}_1$ and $\text {Htr}$ as the expected error of estimating the total slope over a target cohort, the expected regret of not identifying a sub cohort that benefits from the treatment, and whether the total treatment effect heterogeneity presents in the cohort, respectively.

The remaining 8 settings are designed to evaluate the model’s performance in terms of power in two settings, where the HTE is subtle: (1) a non-trivial subgroup of patients is harmed by the treatment, and (2) all patients benefit from the treatment, but the treatment effect is uniformly close to zero. Specifically, in settings 9–12, we set the true HTE to be relatively small with a negative conditional average treatment effect in a subgroup of patients. In settings 13–16, the true treatment effect is uniformly positive but moderate. Detailed descriptions of all 16 settings are provided in the Supplementary File, with an overview available in Table 3.

**Table 3:** A summary of the simulation settings for the 16 settings. ST: step function; CON: continuous function; CST: constant function; IC: informative censoring; NO: without informative censoring; YES: with informative censoring

Setting	$\tau _0(x)$	$\alpha _0(x)$	$\beta _0(x)$	**IC**	Setting	$\tau _0(x)$	$\alpha _0(x)$	$\beta _0(x)$	**IC**
1	ST	ST	ST	NO	9	ST	CST	CST	NO
2	ST	CON	CON	NO	10	CON	CST	CST	NO
3	ST	ST	ST	YES	11	ST	CST	CST	YES
4	ST	CON	CON	YES	12	CON	CST	CST	YES
5	CON	ST	ST	NO	13	CST	CST	CST	NO
6	CON	CON	CON	NO	14	CON	CST	CST	NO
7	CON	ST	ST	YES	15	CST	CST	CST	YES
8	CON	CON	CON	YES	16	CON	CST	CST	YES

$\tau _0(\cdot )$: True conditional average treatment effect on the total eGFR slope

$\alpha _0(\cdot )$: Expected conditional baseline eGFR (intercept)

$\beta _0(\cdot )$: Expected conditional acute eGFR slope

To study the performance of our model with increasing sample sizes, we select total sample sizes *n* of 800 and 2,000 for each setting. For settings 1-8, we compare the treatment recommendation strategy based on our model with a naive strategy that treats all patients. Specifically, we compare our strategy, where only patients with a positive estimated conditional average treatment effect receive treatment (the loss is denoted by $R_{1;propose}$), versus the strategy treating all patients (the loss is denoted by $R_{1;naive}$). The results are presented in Table 4.

**Table 4:** The evaluation of the proposed model under setting 1-8, in which treatment effect heteroeneity is strong. The results are presented in the format of the mean (standard deviation) for $\ell _1$, $R_{1;propose}$, $R_{1;naive}$ and $\text {Htr}$ over 100 Monte Carlo replications for each setting

Setting	*n*	$\ell _1$	$R_{1;propose}$	$R_{1;naive}$	$\text {Htr}$
1	800	0.23 (0.10)	0.01 (0.01)	0.42 (0.03)	0.00 (0.00)
	2,000	0.12 (0.06)	0.01 (0.01)	0.42 (0.02)	0.00 (0.00)
2	800	0.19 (0.09)	0.01 (0.02)	0.42 (0.03)	0.00 (0.00)
	2,000	0.13 (0.06)	0.01 (0.01)	0.42 (0.02)	0.00 (0.00)
3	800	0.25 (0.12)	0.01 (0.01)	0.42 (0.02)	0.00 (0.00)
	2,000	0.13 (0.08)	0.01 (0.01)	0.42 (0.02)	0.00 (0.00)
4	800	0.19 (0.10)	0.02 (0.02)	0.42 (0.02)	0.00 (0.00)
	2,000	0.14 (0.07)	0.01 (0.01)	0.42 (0.02)	0.00 (0.00)
5	800	0.70 (0.06)	0.11 (0.02)	0.61 (0.05)	0.00 (0.00)
	2,000	0.63 (0.03)	0.11 (0.01)	0.60 (0.03)	0.00 (0.00)
6	800	0.69 (0.05)	0.11 (0.02)	0.61 (0.05)	0.00 (0.00)
	2,000	0.62 (0.03)	0.11 (0.01)	0.60 (0.03)	0.00 (0.00)
7	800	0.71 (0.07)	0.11 (0.02)	0.61 (0.05)	0.00 (0.00)
	2,000	0.63 (0.03)	0.11 (0.01)	0.60 (0.03)	0.00 (0.00)
8	800	0.71 (0.05)	0.11 (0.02)	0.61 (0.05)	0.00 (0.00)
	2,000	0.63 (0.02)	0.11 (0.01)	0.60 (0.03)	0.00 (0.00)

$\ell _1$: Mean absolute error in estimating the total treatment effect (lower is better)

$R_1$: Regret of posterior-based treatment decisions relative to the oracle optimum (lower is better)

$\text {Htr}$: Indicator of heterogeneity misclassification (0 = correct, 1 = incorrect; lower is better)

Across all settings above, regardless of the presence of informative censoring, our model consistently demonstrates more accurate estimation of the true total treatment effect as a function of covariates with increasing sample sizes. This is especially evident in cases where the true treatment effect is a step function (settings 1-4), which is consistent with our model specification. On the other hand, in scenarios when our model is mis-specified (settings 5-8), the $\ell _1$ metric decreases slowly since a decision tree (step function) can only approximate a continuous function at a slow rate. Furthermore, the columns $R_{1;propose}$ and $R_{1;naive}$ suggest when a subgroup of the patients indeed has a negative treatment effect, our proposed method effectively reduces the loss compared to the strategy that treats all patients.

Next, we investigate the sensitivity of our model using the remaining settings. For simplicity, we assume that HTE exists for the treatment effect on the total slope, while the intercept, $\alpha _0(\cdot ),$ and the chronic slope, $\gamma _0(\cdot ),$ in the control group remain constant across the covariate space. To objectively calibrate the difficulty of each setting, we define an oracle estimator, named “Bayesian empirical power” (BEP), as follows,

10 $$\begin{aligned} \text {BEP} = \text {Pr}\left(\hat{\tau }_0^{+} - \hat{\tau }_0^{-}> 0\mid \text {Data}\right), \end{aligned}$$

where the probability is with respect to $(\hat{\tau }_0^{+}, \hat{\tau }_0^{-})$ representing posterior distributions for the treatment effect on the total slope obtained by fitting conventional SP models on data subsets where $\tau _0(x_i)> 0$ and $\tau _0(x_i) \le 0$. Observing a high $\text {BEP}$ value serves as an indicator of the signal level of accurately subgrouping patients into those who benefit from the treatment and those who do not. In settings 9 - 12, we set the heterogeneity to be subtle, with $\tau _0^{+} - \tau _0^{-}=0.4$, where $\tau _0^{+}$ and $\tau _0^{-}$ are the treatment effects in patients whose $\tau _0(x)>0$ and those whose $\tau _0(x)\le 0,$ respectively. In settings 14 and 16, the heterogeneity is extremely subtle, with $\tau _0^{+} -\tau _0^{-} =0.2.$ We assume no heterogeneity exists in settings 13 and 15, where $\tau _0^{+} = 0.1$, indicating the treatment effect is 0.1 across the studied population. In the last setting, we set $\hat{\tau }_0^{-}=0$ in BEP, since there is no patient with a negative $\tau _0(x).$ The results for settings 9-16 with $\text {BEP}$ are reported in Table 5,

**Table 5:** The evaluation of the proposed model under settings 9-16, in which the treatment effect heterogeneity is subtle. The results are presented in the format of the mean (standard deviation) for $\ell _1$, $R_{1;propose}$, $R_{1;naive}$ and $\text {Htr}$ over the 100 Monte Carlo replications for each setting

Setting	*n*	$\ell _1$	$R_{1;propose}$	$R_{1;naive}$	BEP	$\text {Htr}$
9	800	0.20 (0.02)	0.10 (0.02)	0.10 (0.01)	0.90 (0.15)	0.96 (0.20)
	2,000	0.14 (0.07)	0.06 (0.05)	0.10 (0.00)	0.98 (0.07)	0.54 (0.50)
10	800	0.25 (0.04)	0.11 (0.04)	0.13 (0.01)	0.93 (0.13)	0.88 (0.33)
	2,000	0.17 (0.06)	0.04 (0.06)	0.13 (0.00)	1.00 (0.01)	0.32 (0.47)
11	800	0.20 (0.03)	0.10 (0.02)	0.10 (0.01)	0.88 (0.17)	0.97 (0.17)
	2,000	0.15 (0.07)	0.06 (0.05)	0.10 (0.00)	0.98 (0.05)	0.64 (0.49)
12	800	0.25 (0.04)	0.12 (0.03)	0.13 (0.01)	0.94 (0.11)	0.92 (0.27)
	2,000	0.19 (0.06)	0.05 (0.06)	0.13 (0.01)	0.98 (0.05)	0.39 (0.49)
13	800	0.08 (0.06)	0.02 (0.04)	0.00 (0.00)	0.75 (0.23)	0.00 (0.00)
	2,000	0.06 (0.04)	0.01 (0.03)	0.00 (0.00)	0.83 (0.20)	0.00 (0.00)
14	800	0.10 (0.04)	0.02 (0.04)	0.00 (0.00)	0.75 (0.24)	1.00 (0.00)
	2,000	0.08 (0.02)	0.01 (0.03)	0.00 (0.00)	0.84 (0.20)	1.00 (0.00)
15	800	0.08 (0.06)	0.02 (0.04)	0.00 (0.00)	0.73 (0.23)	0.00 (0.00)
	2,000	0.06 (0.04)	0.01 (0.03)	0.00 (0.00)	0.81 (0.21)	0.00 (0.00)
16	800	0.10 (0.05)	0.02 (0.04)	0.00 (0.00)	0.73 (0.22)	0.99 (0.10)
	2,000	0.08 (0.02)	0.01 (0.03)	0.00 (0.00)	0.81 (0.19)	1.00 (0.10)

Heterogeneity is subtle in settings 9–12, extremely subtle in setting 13 and 15, and absent in settings 14 and 16

$\ell _1$: Mean absolute error in estimating the total treatment effect (lower is better)

$R_1$: Regret of posterior-based treatment decisions relative to the oracle optimum (lower is better)

$\text {Htr}$: Indicator of heterogeneity misclassification (0 = correct, 1 = incorrect; lower is better)

Our model faces challenges to capture the underlying HTE when the sample size is relatively small, i.e., $n = 800$. This is evident from the similar values observed in columns $R_{1;propose}$ and $R_{1;naive}$, as well as $\text {Htr}$ values approaching 1 in settings 9 - 12. These patterns suggest our model produces results similar to the simple strategy that treats all patients uniformly. Despite the $\text {BEP}$ values being close to 1, indicating a strong signal of between-subgroup differences from a Bayesian perspective given the accurate subgrouping, our model struggles to detect these differences. This limitation is understandable because estimation based on the subgrouping identified through decision trees is more complex than the one given the true subgrouping information and subjects to estimation error. As the sample size increases to $n = 2,000$, our model detects the HTE, indicated by the reduced values in columns $R_{1;propose}$ and $\text {Htr}$. However, in settings 14 and 16, the heterogeneity remains too subtle to be detected, even an with increased sample size. The low $\text {BEP}$ values suggest the chosen sample size is limited, which may explain why our model cannot satisfactorily capture the heterogeneity, as evidenced by $\text {Htr}$ values close to 1.

Our model also demonstrates its ability to avoid overfitting, as evidenced by the results from settings 13 and 15. In general, Bayesian decision trees naturally impose a strong regularization on splitting small nodes through prior elicitation, unlike Frequentist methods, which may spuriously detect false positive subgroups by focusing on within‑leaf variance [30]. Consistent with this, our Bayesian model produced no subgroups when no true or only weak HTE was present, as indicated by the corresponding $\textrm{Htr}$ values. These findings, along with previous results, suggest that our model will only detect and report the presence of sufficiently strong HTE. They also support using $\mathbbm{1}(\hat{\tau }_{0}(x_1) = \ldots = \hat{\tau }_{0}(x_{n_2}))$ as a reference for confirming HTE in subsequent real‑data analyses.

## MDRD data analysis

In this section, we apply our method to reanalyze the Modification of Diet in Renal Disease study (MDRD; [18, 19]). The MDRD study consists of two randomized clinical trials that investigated whether protein restriction and/or blood pressure control have an effect on the progression of CKD. In study 1, 585 patients with GFR of 25-55 $ml/min/1.73~m^2$ were randomly assigned to a usual-protein diet or a low-protein diet (1.3 or 0.58 g of protein per kilogram of body weight per day) and to a usual- or a low-blood-pressure group (mean arterial pressure, 107 or 92 mm Hg). In study 2, 255 patients with GFR of 13 to 24 *ml*/*min*/1.73 *m*2 were randomly assigned to the low-protein diet (0.58 g per kilogram per day) or a very-low-protein diet (0.28 g per kilogram per day) with a keto acid-amino acid supplement, and a usual- or a low-blood-pressure group (same values as those in study 1). The length of follow-up varied from 18 to 45 months, with monthly evaluations of the patients.

Here we quantify heterogeneous treatment effects of a “usual” versus low mean arterial blood pressure (MAP) target (102 v. 92 mm Hg) in delaying the progression of CKD. The treatment effect of interest is the difference in three-year total eGFR slopes between the treatment (low MAP) and control (high MAP) groups. The time to event outcome censoring the eGFR trajectory have been described in “A shared parameter model structure for informative censoring” section. To characterize the treatment effect heterogeneity with interpretable clinical decision rules, we will use the covariates described in Table 6, where the $\log (1+x)$ transformation is applied to continuous covariates to mitigate the heavy-tail of the distribution. The final sample size for analysis is reduced to 815 from 840, after removing the samples with missing covariates listed in Table 6.

**Table 6:** Baseline covariates of MDRD patients and their definition

Type	Name	Definition
Continuous	eGFR	Estimated Glomerular Filtration Rate (eGFR)
	AGE	Age (year)
	UACR	Urine Albumin/Creatinine Ratio (mg/g)
	PHOS	Serum Phosphorus (mg/dl)
	ALB	Albumin (g/dl)
	BICARB	Bicarbonate (Meq-L)
	BMI	Body Mass Index (kg/$\text {m}^2$)
	CAL	Calcium (Mg-Dl)
	DBP_sit	Diastolic blood pressure measured in the sitting position (mmHg)
	SBP_sit	Systolic blood pressure measured in the sitting position (mmHg)
	LDL	LDL Serum Cholesterol (mg/dl)
	HDL	HDL Serum Cholesterol (mg/dl)
	TG	Triglycerides (mg/dl)
Discrete	Sex	Male = 0; Female = 1
	Smoke	No = 0; Yes = 1
	Race	Hispanic = 0; Non-Hispanic black = 1;
		Non-Hispanic white = 2; Other = 3

Note that in our within-leaf linear regressions, defined in (7), we regress the control chronic slope $\gamma _0(\cdot )$ and the total treatment effect $\tau _0(\cdot )$ on baseline eGFR, AGE, UACR and PHOS. The intercept $\alpha _0(\cdot )$, representing the expected baseline eGFR, is regressed on baseline AGE, UACR and PHOS only.

**Table 7:** Within-subgroup total slopes for both the treatment and control groups are shown as the posterior means. Treatment effects are presented as the mean (95$\%$ credible interval), based on 12,500 posterior samples. The results presented are derived from the 40$\%$ (validation) and 60$\%$ (training) data subsets, respectively

Part	Group	*E*(*n*)[*E*(*p*)]	Control slope	Treatment slope	Total treatment effect
$40\%$	1	146.30[0.05]	−3.54	−2.44	1.10 ([−0.41,2.39])
(Validation)	2	180.10[0.32]	−4.27	−4.00	0.27 ([−0.97,1.41])
$60\%$	1	218.23[0.05]	−3.49	−2.50	0.99 ([−0.41,2.39])
(Training)	2	270.18[0.32]	−4.26	−4.05	0.21 ([−0.97,1.41])

**Fig. 2:**
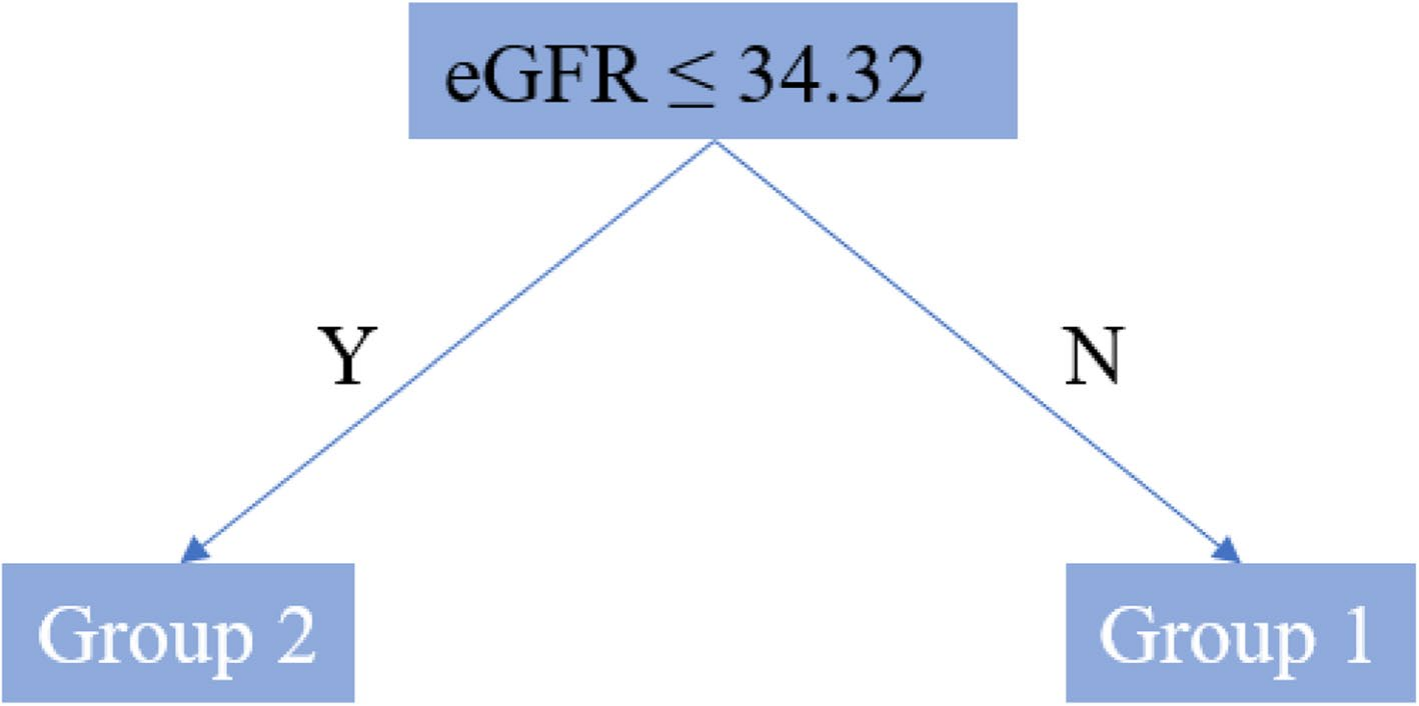
The “most representative tree” on the total treatment effect $\tau _0(x)$ obtained in the MDRD study

**Fig. 3:**
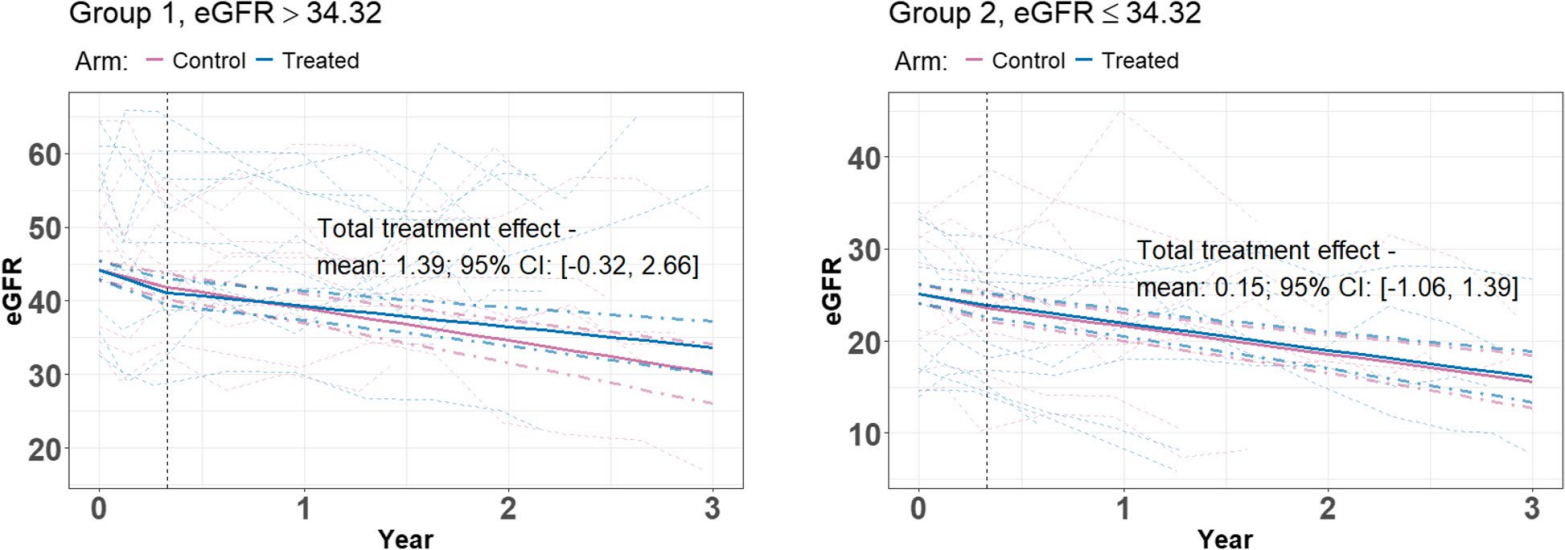
Spaghetti plot illustrating the group-wise mean trends, with dot-dashed lines representing the 95$\%$ credible bands, based on sub-sampled data for each treatment arm. The dashed line marks the boundary between the acute and chronic stages within the three-year observation period

Using the prior specification in “Bayesian hierarchical structure” section and the training procedure introduced in “Implementation” section, our model generates “most representative tree”, based on $B_2=100$ posterior samples after 100 warm-up iterations. Across $B_3=100$ markov chains with different data splittings, we identify 100 “most representative trees” from different discovery sets, and present a “super-representative tree” with the highest frequency ($48\%$) in Fig. 2. Another tree with a frequency of $43\%$ is provided in the Supplementary File. The same “most representative tree” may appear multiple times because only pre-specified cut offs are used in constructing those trees. The detailed within-subgroup treatment effect inferences, shown in Table 7, are based on $48\times 1000$ posterior samples generated from the presented tree structure. These samples are obtained by collecting 1,000 posterior samples after 1,000 warm-up iterations from the $40\%$ data, i.e., the validation set, considering only samples from data splitting that yields the “super-representative tree” in Fig. 2. The sample size of each group and the proportion of uncensored time to event outcomes is reported as average values over these 48 Markov chains, denoted by *E*(*n*)[*E*(*p*)]. In Fig. 3, we graphically present inference results on eGFR trajectories in two subgroups identified by the “super-representative tree”. Additionally, we show the group-wise spaghetti plot using 20 trajectories for each identified subgroup, with a random sample of 10 trajectories representing each arm of the study.

Based on the 100 “most representative trees”, we identify strong evidence of HTE on the total eGFR slope, supported by the fact that all “most representative trees” have at least two leaves. However, it is noteworthy that all the credible intervals above the middle line in Table 7 are overlapping, suggesting a potential lack of statistically significant evidence of HTE from a frequentist perspective. To address this concern, we provide additional evidence for its presence via a Bayesian approach.

From these 48,000 posterior samples, we observe that the treatment is significantly more effective in Group 1 than in Group 2 with a posterior frequency of 81%. It is important to recognize that this evidence is derived from only 40$\%$ of the data, i.e., validation sets from 48 data splittings, and the within-leaf linear model defined in “Heterogeneous treatment effect detection with Bayesian decision trees” section may lead to overfitting due to limited sample sizes. To obtain more reliable result and stronger evidence, additional replication in a larger independent dataset is required. In Table 8, the within-subgroup baseline covariates are presented to better characterize the groups identified.

**Table 8:** Within-subgroup baseline covariates of MDRD patients, presented as median (interquartile range) for continuous variables and mean (standard deviation) for binary variable, in the entire dataset. Total treatment effect values on the 3-year eGFR slope are dervied from the 40$\%$ testing sample only.

Covariates		Group	
Type	Name	1 ($\text {eGFR}> 34.32$)	2 ($\text {eGFR} \le 34.32$)
Continuous	eGFR	44.23 (11.62)	23.97 (11.48)
	AGE	52.00 (18.00)	53.00 (21.00)
	UACR	78.53 (212.15)	194.83 (293.31)
	PHOS	3.50 (0.70)	4.00 (0.90)
	ALB	4.10 (0.50)	4.00 (0.40)
	BICARB	24.00 (4.25)	22.00 (4.00)
	BMI	27.29 (5.42)	26.04 (5.84)
	CAL	9.10 (0.50)	9.00 (0.70)
	DBP_sit	81.00 (14.25)	81.00 (12.00)
	SBP_sit	130.00 (22.00)	131.00 (23.00)
	LDL	144.00 (56.00)	144.00 (53.50)
	HDL	37.00 (17.00)	37.00 (18.00)
	TG	147.00 (125.00)	135.00 (101.50)
Binary	if Female	0.37 (0.49)	0.41 (0.49)
	if Hispanic	0.04 (0.19)	0.05 (0.21)
	if non-Hispanic black	0.08 (0.27)	0.07 (0.26)
	if non-Hispanic white	0.86 (0.35)	0.86 (0.34)
	if other	0.02 (0.15)	0.02 (0.12)
Total treatment effect		1.10 ([−0.41,2.39])	0.27 ([−0.97,1.41])

**Fig. 4:**
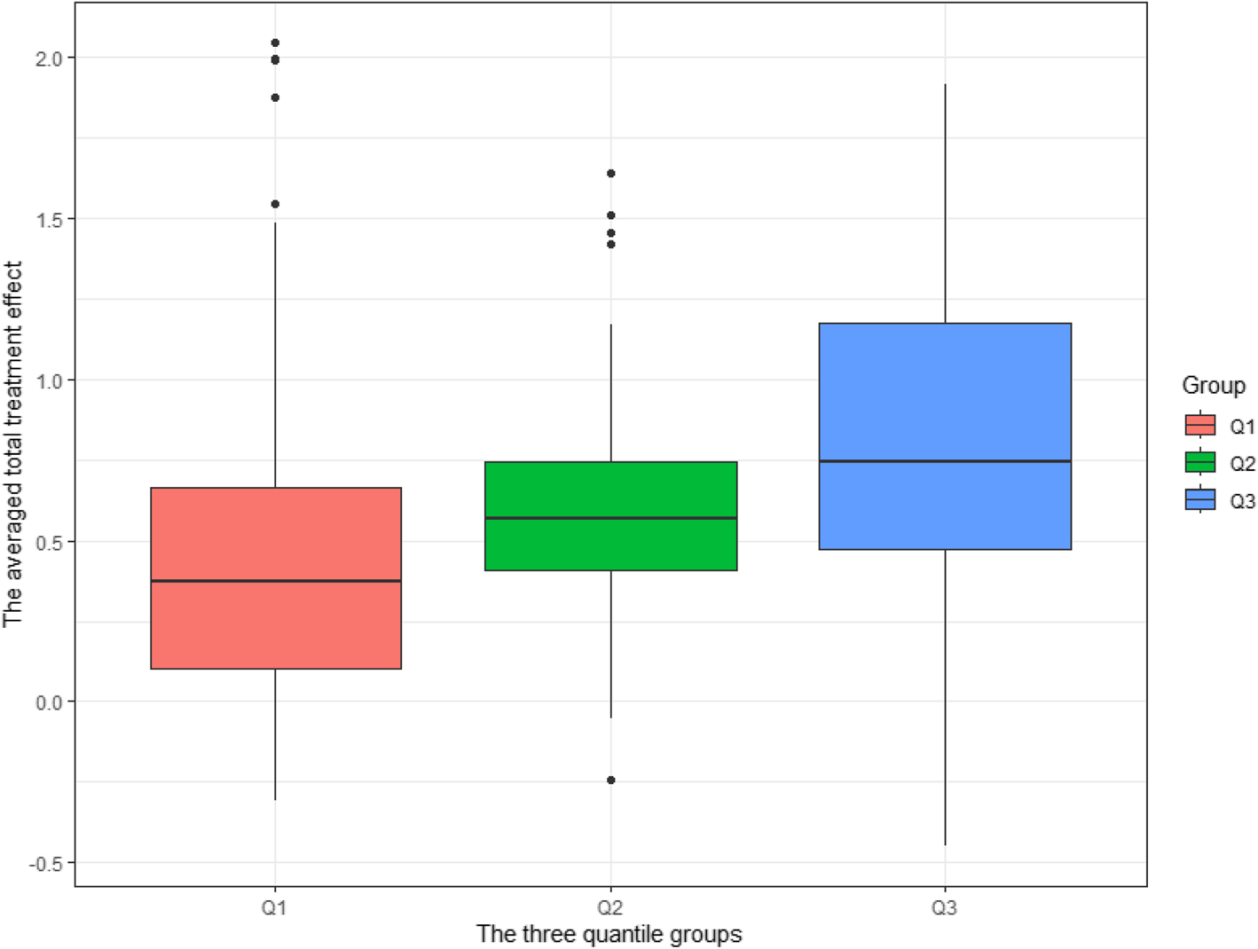
Assessment of the concordance between the two data subsets (60/40 split) across the 100 ’minimum distance trees.’ Each box represents the distribution of individual treatment effects estimated on the validation set (60% subset), conditioned on each subgroup identified from the discovery set (40% subset). The three subgroups are defined by evenly spaced quantiles of individual treatment effects calculated from the discovery set

Our model demonstrates a satisfactory reproducibility in two ways. First, Table 7 demonstrates that the subgroup-specific treatment effects estimated from the discovery and validation sets are consistent, for the single most representative tree. This consistency indicates that our model produces similar results across data used for tree searching/development and data used for treatment effect estimation. Second, we evaluate the concordance in the treatment effect estimates between these two data subsets across $B_3=100$ random data splits, in which 100 “most representative trees” are generated. For each data split, patients in the validation set are sorted and grouped into three subgroups of equal sizes according to their conditional average treatment effect estimates obtained from the most representative tree in the discovery set. The treatment effect estimates derived from the validation sets for patients in each subgroup are then averaged to approximate the group-specific treatment effect. The distribution of group-specific treatment effect estimates is visualized using a boxplot in Fig. 4, which shows that the group-specific treatment effect estimates based on validation sets indeed tend to be concordant with “most representative trees” constructed on discovery sets.

## Discussion

In this paper, we have introduced a novel method that simultaneously estimates HTE on the eGFR slope using a BDT and addresses potential informative censoring through a two-slope shared parameter (SP) model. Our simulation results suggest that the proposed model can detect and estimate HTEs of interest. We have demonstrated the utility of our approach as applied to the MDRD trial.

Technically, there are four main challenges that require further investigation. First, the current sampling algorithm is time-consuming, primarily due to its reliance on the Gibbs sampler. The major time consumption arises from sampling tree structures. To enhance overall sampling efficiency, one could explore variational Bayes alternatives for sampling trees [31]. Second, as discussed by [1], the SP model might encounter convergence issues due to its two-slope structure. One possible reason is that the estimation of the acute slope heavily depends on early-stage data, where observed eGFR levels only provide little information. This poses difficulties in inferring HTEs on the total eGFR slope, which is a linear combination of the acute and chronic slopes. To Address this issue, one may leverage additional information sources to accurately estimate the acute eGFR slope. Third, the current data-splitting procedure reduces the effective sample size for both HTE discovery and downstream analyses. A potential solution is to integrate these two steps by accounting for bias resulting from tree splitting. The selective inference procedure introduced by offers a possible approach [32], though it still requires thorough investigation in the context of the SP model. Fourth, the model may generate spurious HTE signals for the total treatment effect if heterogeneity in the control-arm chronic slope is not properly accounted for. This risk is amplified in high-dimensional settings, where small sample sizes within each leaf node limit our ability to estimate $\gamma _{0}(x)$ accurately via within-leaf linear regressions. As a result, residual variation in the control slope can be absorbed into the treatment-effect estimates, producing false signals of heterogeneity. A straightforward remedy is to employ high-dimensional regression techniques—such as Bayesian realizations of lasso or elastic net to ensure robust estimation of $\gamma _{0}(x)$ before assessing $\tau _0(x)$.

Increasing the power of inference is also an important area for future work. As shown in “MDRD data analysis” section, the credible intervals for the identified subgroups overlap substantially, indicating non-significant heterogeneity from a frequentist perspective, but strong evidence for the existence of heterogeneity from the Bayesian perspective. There are several possible directions to mitigate this issue. First, the total treatment effect involves the acute treatment effect, which is difficult to estimate. This may contribute to the large variance in the total treatment effect. Redesigning the parametric model for the acute slope might help alleviate this problem. Second, to increase power, one could incorporate biological constraints, such as enforcing that individual eGFR trajectories are monotonic.

In summary, we have developed a Bayesian method that can flexibly identify interpretable subgroups of patients with demonstrated treatment effect heterogeneity on the total eGFR slope.

## Supplementary Information

Supplementary Material 1.

## Data Availability

The MDRD data are available from the NIDDK central biorepository upon request: https://repository.niddk.nih.gov/studies/mdrd/.

## References

[1] Vonesh E, Tighiouart H, Ying J, Heerspink HL, Lewis J, Staplin N, et al. Mixed-effects models for slope-based endpoints in clinical trials of chronic kidney disease. Stat Med. 2019;38(22):4218–39. 31338848 10.1002/sim.8282

[2] Schoenfeld DA. Sample-size formula for the proportional-hazards regression model. Biometrics. 1983. 10.2307/2531021 6354290

[3] Inker LA, Heerspink HJ, Tighiouart H, Levey AS, Coresh J, Gansevoort RT, et al. Gfr slope as a surrogate end point for kidney disease progression in clinical trials: a meta-analysis of treatment effects of randomized controlled trials. J Am Soc Nephrol. 2019;30(9):1735–45. 31292197 10.1681/ASN.2019010007 PMC6727261

[4] Grams ME, Sang Y, Ballew SH, Matsushita K, Astor BC, Carrero JJ, et al. Evaluating glomerular filtration rate slope as a surrogate end point for ESKD in clinical trials: an individual participant meta-analysis of observational data. J Am Soc Nephrol. 2019;30(9):1746–55. 31292199 10.1681/ASN.2019010008 PMC6727262

[5] Lysaght MJ, Vonesh EF, Gotch F, Ibels L, Keen M, Lindholm B, et al. The influence of dialysis treatment modality on the decline of remaining renal function. ASAIO J. 1991;37(4):598–604. 1768496

[6] Moist LM, Port FK, Orzol SM, Young EW, Ostbye T, Wolfe RA, et al. Predictors of loss of residual renal function among new dialysis patients. J Am Soc Nephrol. 2000;11(3):556–64. 10703680 10.1681/ASN.V113556

[7] Vonesh EF, Greene T, Schluchter MD. Shared parameter models for the joint analysis of longitudinal data and event times. Stat Med. 2006;25(1):143–63. 16025541 10.1002/sim.2249

[8] Hogan JW, Roy J, Korkontzelou C. Handling drop-out in longitudinal studies. Stat Med. 2004;23(9):1455–97. 15116353 10.1002/sim.1728

[9] Wu MC, Carroll RJ. Estimation and comparison of changes in the presence of informative right censoring by modeling the censoring process. Biometrics. 1988. 10.2307/2531905

[10] Collier W, Inker LA, Haaland B, Appel GB, Badve SV, Caravaca-Fontán F, et al. Evaluation of variation in the performance of GFR slope as a surrogate end point for kidney failure in clinical trials that differ by severity of CKD. Clin J Am Soc Nephrol. 2023;18(2):183–92. 36754007 10.2215/CJN.0000000000000050 PMC10103374

[11] Inker LA, Collier W, Greene T, Miao S, Chaudhari J, Appel GB, et al. A meta-analysis of GFR slope as a surrogate endpoint for kidney failure. Nat Med. 2023;29(7):1867–76. 37330614 10.1038/s41591-023-02418-0 PMC13037386

[12] Kent DM, Steyerberg E, Van Klaveren D. Personalized evidence based medicine: predictive approaches to heterogeneous treatment effects. BMJ. 2018;363. 10.1136/bmj.k4245 PMC6889830 30530757

[13] Su X, Meneses K, McNees P, Johnson WO. Interaction trees: exploring the differential effects of an intervention programme for breast cancer survivors. J R Stat Soc Ser C Appl Stat. 2011;60(3):457–74.

[14] Wei Y, Liu L, Su X, Zhao L, Jiang H. Precision medicine: subgroup identification in longitudinal trajectories. Stat Methods Med Res. 2020;29(9):2603–16. 32070237 10.1177/0962280220904114 PMC8357634

[15] Yang J, Mwangi AW, Kantor R, Dahabreh IJ, Nyambura M, Delong A, et al. Tree-based subgroup discovery using electronic health record data: heterogeneity of treatment effects for DTG-containing therapies. Biostatistics. 2024;25(2):323–35. 37475638 10.1093/biostatistics/kxad014 PMC11017113

[16] Chipman HA, George EI, McCulloch RE. Bayesian CART model search. J Am Stat Assoc. 1998;93(443):935–48.

[17] Chipman HA, George EI, McCulloch RE. Bayesian treed models. Mach Learn. 2002;48:299–320.

[18] Beck G, Berg R, Coggins C, Gassman J, Hunsicker L, Schluchter M, et al. Design and statistical issues of the Modification of Diet in Renal Disease Trial. The Modification of Diet in Renal Disease Study Group. Control Clin Trials. 1991;12(5):566–86. 10.1016/0197-2456(91)90069-x 1664792

[19] Klahr S, Levey AS, Beck GJ, Caggiula AW, Hunsicker L, Kusek JW, et al. The effects of dietary protein restriction and blood-pressure control on the progression of chronic renal disease. N Engl J Med. 1994;330(13):877–84. 8114857 10.1056/NEJM199403313301301

[20] Follmann D, Wu M. An approximate generalized linear model with random effects for informative missing data. Biometrics. 1995:151–68. 7766771

[21] Friedman M. Piecewise exponential models for survival data with covariates. Ann Stat. 1982;10(1):101–13.

[22] Berger JO, Wang X, Shen L. A bayesian approach to subgroup identification. J Biopharm Stat. 2014;24(1):110–29. 24392981 10.1080/10543406.2013.856026

[23] van Klaveren D, Balan TA, Steyerberg EW, Kent DM. Models with interactions overestimated heterogeneity of treatment effects and were prone to treatment mistargeting. J Clin Epidemiol. 2019;114:72–83. 31195109 10.1016/j.jclinepi.2019.05.029 PMC7497896

[24] Guo J, Gabry J, Goodrich B, Weber S. Package ‘rstan’. 2020. https://cloud.r-project.org/web/packages/rstan/index.html

[25] Hoffman MD, Gelman A, et al. The No-U-Turn sampler: adaptively setting path lengths in Hamiltonian Monte Carlo. J Mach Learn Res. 2014;15(1):1593–623.

[26] Heled J, Bouckaert RR. Looking for trees in the forest: summary tree from posterior samples. BMC Evol Biol. 2013;13:1–11. 24093883 10.1186/1471-2148-13-221 PMC3853548

[27] Athey S, Imbens G. Recursive partitioning for heterogeneous causal effects. Proc Natl Acad Sci U S A. 2016;113(27):7353–60. 27382149 10.1073/pnas.1510489113 PMC4941430

[28] Wager S, Athey S. Estimation and inference of heterogeneous treatment effects using random forests. J Am Stat Assoc. 2018;113(523):1228–42.

[29] Rizopoulos D. Dynamic predictions and prospective accuracy in joint models for longitudinal and time-to-event data. Biometrics. 2011;67(3):819–29. 21306352 10.1111/j.1541-0420.2010.01546.x

[30] Breiman L, Friedman J, Olshen RA, Stone CJ. Classification and regression trees. Routledge; 2017.

[31] Blei DM, Kucukelbir A, McAuliffe JD. Variational inference: a review for statisticians. J Am Stat Assoc. 2017;112(518):859–77.

[32] Neufeld AC, Gao LL, Witten DM. Tree-values: selective inference for regression trees. J Mach Learn Res. 2022;23(305):1–43. PMC10933572 38481523

