# Supplementary material for: Discovering heterogeneous treatment effects on slope-based endpoints in chronic kidney disease trials
Source: BMC Med Res Methodol. 2025 Oct 14;25:230. doi: 10.1186/s12874-025-02646-7 (PMC12523132; doi:10.1186/s12874-025-02646-7)

# Supplementary File for "Discovering Heterogeneous Treatment Effects on Slope-based Endpoints in Chronic Kidney Disease Trials"

Tianyu Pan[1,2], Lu Tian[1], Manjula Kurella Tamura[3,4], Maria Montez-Rath[3], Vivek Charu[2,5*]

[1]Department of Biomedical Data Science, Stanford University School of Medicine, 1265 Welch Road, Stanford, 94305, CA, USA.
[2]Department of Pathology, Stanford University School of Medicine, 300 Pasteur Drive, Stanford, 94305, CA, USA.
[3]Division of Nephrology, Department of Medicine, Stanford University School of Medicine, 3180 Porter Drive, Palo Alto, 94304, CA, USA.
[4]Veterans Affairs Palo Alto Health Care System, 3801 Miranda Avenue, Palo Alto, 94304, CA, USA.
[5]Quantitative Sciences Unit, Department of Medicine, Stanford University School of Medicine, 300 Pasteur Drive, Stanford, 94305, CA, USA.

*Corresponding author(s). E-mail(s): vcharu@stanford.edu;
Contributing authors: tianyup2@stanford.edu; lutian@stanford.edu; mktamura@stanford.edu; mmrath@stanford.edu;

**Abstract**

This file is organized as follows. In Section 1, the detailed DGPs are listed into a summary table. In Section 2, we represent another representative tree mentioned in Section 5 in the main article. The detail of generating intersection trees is provided in Section 3. The comparison between our model's results with the existing ones is presented in Section 4. The diagnosis of incorporating a shared-parameter model structure is detailed in Section 5. The implementation pipeline is illustrated in Section 6.

# 1 Details of the DGPs

In this section, we present the details of the DGPs used in the main article. For notation clarity, we let $x^{(j)}$ denote the $j$-th entry of row vector $x$, to distinguish from the notation $x_i$, which denotes a row vector of length $p$, the baseline covariate of the $i$-th subject. Additionally, we let $x$ and $x_i$ be the realization of random vectors $\boldsymbol{x}$ and $\boldsymbol{x_i}$. We also let $\alpha_0(x)$, $\beta_0(x)$, $\gamma_0(x)$, $\tau_0(x)$ and $\zeta_0(x)$ denote the true function values of the expected baseline eGFR, the acute slope, the chronic slope, the treatment effect on the total and acute slopes, respectively. Following the description in Section 4 of the main article, the generating process of $\boldsymbol{x_i}$ satisfies

$$\begin{aligned} \boldsymbol{x_i}^{(j)} &\overset{i.i.d.}{\sim} \text{Unif}(-2,2), \quad \text{for } i=1,\dots,n, \quad j=1,\dots,p-1, \\ \boldsymbol{x_i}^{(j)} &\overset{i.i.d.}{\sim} \text{Bernoulli}(0.5), \quad \text{for } i=1,\dots,n, \quad j=p, \end{aligned} \tag{1}$$

where $p$ is set to 6 for all 16 DGPs. The DGPs are presented in Table . Note that we choose $\eta_1 = \eta_2 = 0$ for DGP 1, 2, 5, 6, 9, 10, 13, 14 to simulate the case when the censoring is non-informative, and $\eta_1 = \eta_2 = -0.5$ for DGP 3, 4, 7, 8, 11, 12, 15, 16 to mimic the scenarios when patients are more likely to drop out when their eGFR decline more rapidly.

# 2 Another tree discovered in the MDRD dataset

In this section, we present the tree with a posterior frequency of 43%, mentioned in Section 5. The tree indicates three subgroups, and the tree configuration is approximately identical to the one presented in the main article except for an additional split on the left child.

**Table 1**: Details of the 16 DGPs. The treatment effect on the acute slope $\zeta_0(x)$ is assumed to be 0 for all the 16 DGPs.

| DGP | $\alpha_0(x)$ | $\beta_0(x)$ | $\gamma_0(x)$ | $\tau_0(x)$ |
|---|---|---|---|---|
| 1 | $80 + 60 \times \mathbb{1}(x^{(1)} > 0)\times$ | $-5$ | $-1 - 3\times$ | $-1 + 2 \times \mathbb{1}(x^{(1)} > -\frac{1}{3})\times$ |
| 3 | $\left[1 + \mathbb{1}(x^{(6)} = 1)\right]$ | | $\mathbb{1}(x^{(1)} > 0)$ | $\left[1 + \mathbb{1}(x^{(6)} = 1)\right]$ |
| 2 | $80 + 20 \times \cos(\frac{x^{(1)}}{2}\pi)+$ | $-5$ | $-2 - 2\times$ | $-1 + 2 \times \mathbb{1}(x^{(1)} > -\frac{1}{3})\times$ |
| 4 | $40 \times \mathbb{1}(x^{(6)} = 1)$ | | $\sin((\frac{x^{(1)}-1}{2})\pi)$ | $\left[1 + \mathbb{1}(x^{(6)} = 1)\right]$ |
| 5 | $80 + 60 \times \mathbb{1}(x^{(1)} > 0)\times$ | $-5$ | $-1 - 3\times$ | $-1 + 2 \times \mathbb{1}(x^{(6)} = 1)+$ |
| 7 | $\left[1 + \mathbb{1}(x^{(6)} = 1)\right]$ | | $\mathbb{1}(x^{(1)} > 0)$ | $\frac{1}{3} \times \left(x^{(1)}\right)^3$ |
| 6 | $80 + 20 \times \cos(\frac{x^{(1)}}{2}\pi)+$ | $-5$ | $-2 - 2\times$ | $-1 + 2 \times \mathbb{1}(x^{(6)} = 1)+$ |
| 8 | $40 \times \mathbb{1}(x^{(6)} = 1)$ | | $\sin((\frac{x^{(1)}-1}{2})\pi)$ | $\frac{1}{3} \times \left(x^{(1)}\right)^3$ |
| 9 | 80 | $-5$ | $-2$ | $-0.2 + 0.4 \times \mathbb{1}(x^{(6)} = 1)$ |
| 11 | | | | |
| 10 | 80 | $-5$ | $-2$ | $0.4 \times \sin(\frac{x^{(1)}}{2}\pi)$ |
| 12 | | | | |
| 13 | 80 | $-5$ | $-2$ | 0.1 |
| 15 | | | | |
| 14 | 80 | $-5$ | $-2$ | $0.1 + 0.1 \times \sin(\frac{x^{(1)}}{2}\pi)$ |
| 16 | | | | |

**Fig. 1**: Another representative tree obtained in the MDRD study

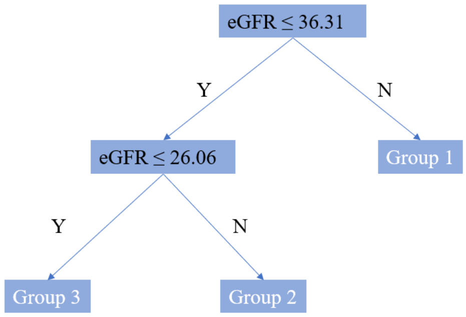


# 3 Producing intersection trees

In this section, we formally present the method introduced in the main article for generating the intersection tree. To generate an intersection tree based on two trees, $Tree_1$ and $Tree_2$, a straightforward approach is to grow $Tree_2$ at each leaf of $Tree_1$, as illustrated in Figure 2.

In the MDRD analysis, we first grow the tree corresponding to the control chronic slope at each leaf of the total treatment effect tree. After obtaining this intersection

**Fig. 2**: The intersection tree of $Tree_1$ and $Tree_2$.

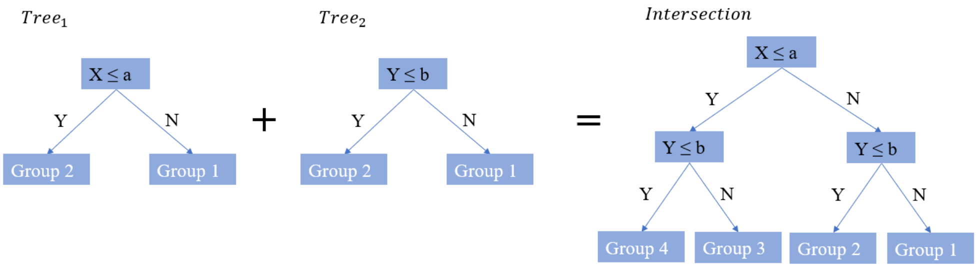


tree, we then grow the tree on the intercept at each leaf of it. Notably, a leaf will not proceed to further tree growing if its size (i.e., the number of subjects in that subgroup) falls blow a certain threshold. In the MDRD study, this threshold is set to 80.

## 4 The Kaplan-Meier estimation and within-group trends obtained from the entire data

In this section, we compare the survival probabilities estimated using the Kaplan-Meier estimator with the within-group trends estimated by the simple model based on the intersection tree, as described in Section 5 of the main article. For the Kaplan-Meier estimator, the survival outcome of interest is the end-stage kidney disease (ESKD) over the entire MDRD study period, consistent with the definition used in the meta-analysis by [1]. Both estimations are based on the full dataset, and the results are presented in Panel 3.

The Kaplan-Meier results suggest that treatment with a lower mean arterial pressure (MAP) goal is ineffective across the entire population and within the identified subgroups. This is supported by the overlapping confidence intervals throughout the study period, as shown in sub-figures (a), (b), and (c), as well as the log-rank test results (p-values - Entire population: 0.4; Group 1, higher baseline eGFR: 0.8; Group

2, lower baseline eGFR: 0.3). These findings mismatch our model's results, which indicate that the treatment is effective in Group 1, the higher baseline eGFR group (i.e., sub-figure (e)). For the entire population, although the 95% credible interval includes 0 for our model, this inclusion may be due to the margin of error. Despite this, the results still provide strong evidence of a positive average treatment effect.

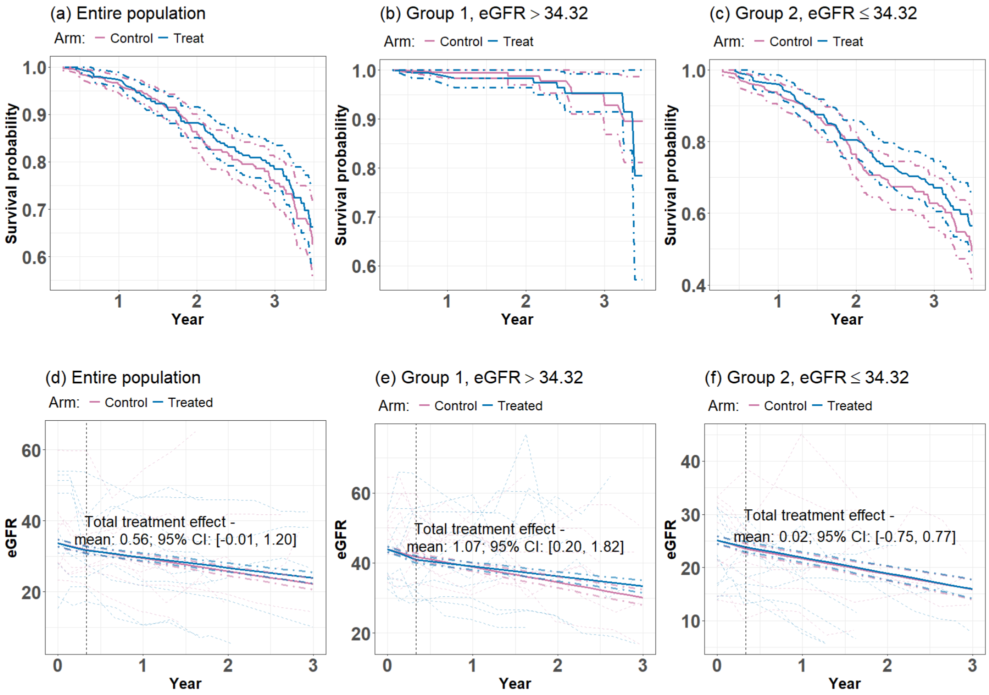


**Fig. 3**: The within-group trends and the event-free survival probabilities given by the Kaplan-Meier estimate. The event represents observing the composite kidney outcome.

# 5 The evidence of the presence of informative censoring

In this section, we diagnose the existence of informative censoring based on our proposed model. Based on the 48,000 posterior samples, the median value and the 95 % credible interval of $\eta_1$ and $\eta_2$ are -0.20 ([-0.37,-0.08]) and -0.65 ([-1.02, -0.43]),

respectively. These findings provide Bayesian evidence for the existence of informative censoring with an interpretation that those patients with lower baseline eGFR and faster decline rates in eGFR exhibit shorter time to the survival endpoints in expectation. The results justifies the use of a shared parameter structure, in addition to the combination of a linear mixed effect model with a Bayesian decision tree.

# 6 The implementation pipeline

In this section, we present the implementation pipeline mentioned in the main article, detailed in Figure 4 and 5.

**Fig. 4**: The implementation pipeline given a data splitting configuration.

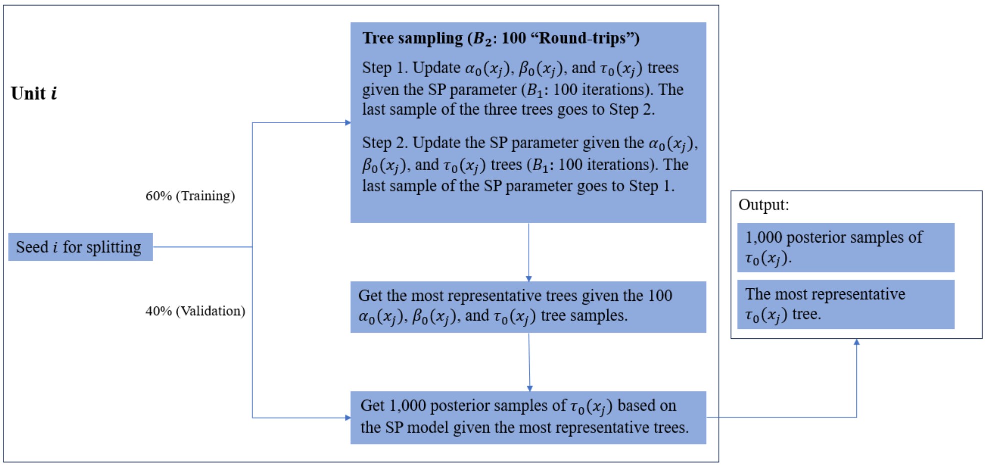


# References


1. Ku E, McCulloch CE, Inker LA, Tighiouart H, Schaefer F, Wühl E, et al. Intensive BP control in patients with CKD and risk for adverse outcomes. Journal of the American Society of Nephrology. 2023;34(3):385–393.

**Fig. 5**: The implementation pipeline of combining the representative trees given different data splitting configurations.

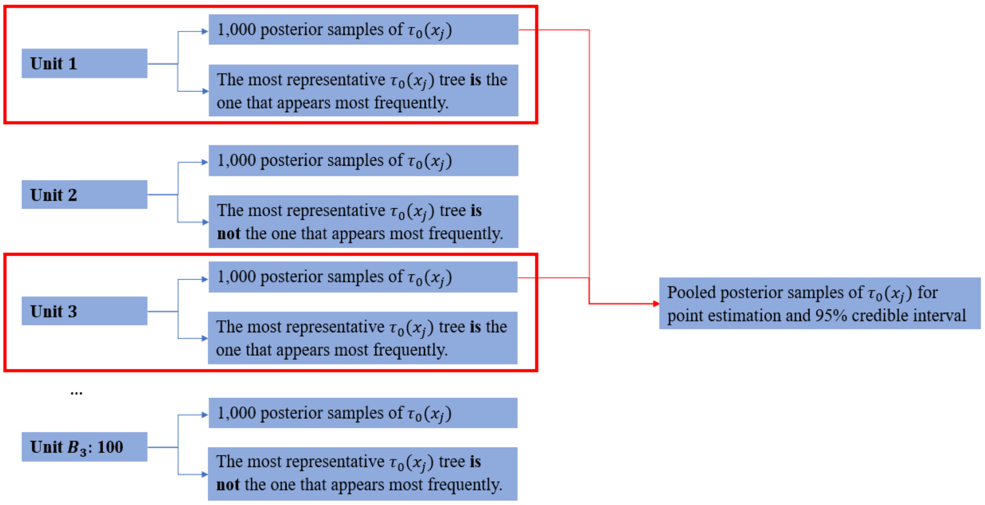

Supplement: Supplementary file 1 — Supplementary Material 1. [file 12874_2025_2646_MOESM1_ESM.pdf]
